# Endocannabinoid signaling regulates post-operative delirium through glutamatergic mediodorsal thalamus-prelimbic prefrontal cortical projection

**DOI:** 10.3389/fnagi.2022.1036428

**Published:** 2022-12-01

**Authors:** Yang Liu, Sansan Jia, Jiajia Wang, Dan Wang, Xinxin Zhang, Huiqing Liu, Fang Zhou, Zhihao Zhang, Qi Li, Hailong Dong, Haixing Zhong

**Affiliations:** Department of Anesthesiology and Perioperative Medicine, Xijing Hospital, The Fourth Military Medical University, Xi'an, China

**Keywords:** post-operative delirium, endocannabinoid signaling, mediodorsal thalamus, type 1 cannabinoid receptor, prelimbic prefrontal cortex

## Abstract

**Background:**

Post-operative delirium (POD), a common post-operative complication that affects up to 73. 5% of surgical patients, could prolong hospital stays, triple mortality rates, cause long-term cognitive decline and dementia, and boost medical expenses. However, the underlying mechanisms, especially the circuit mechanisms of POD remain largely unclear. Previous studies demonstrated that cannabis use might cause delirium-like behavior through the endocannabinoid system (eCBs), a widely distributed retrograde presynaptic neuromodulator system. We also found that the prelimbic (PrL) and intralimbic (IL) prefrontal cortex, a crucial hub for cognition and emotion, was involved in the eCBs-associated general anesthesia recovery.

**Objectives:**

The present study aimed to investigate the role of eCBs in POD development, and further clarify its neuronal specificity and circuit specificity attributed to POD.

**Methods:**

According to a previous study, 2 h of 1.4% isoflurane anesthesia and simple laparotomy were conducted to establish the POD model in C57/BL6 mice aged 8–12 weeks. A battery of behavioral tests, including the buried food, open field, and Y maze tests, were performed at 24 h before anesthesia and surgery (AS) and 6 and 9 h after AS. The behavioral results were calculated as a composite Z score for the POD assessment. To explore the dynamics of eCBs and their effect on POD regulation, an endocannabinoid (eCB) sensor was microinjected into the PrL, and the antagonists (AM281 and hemopressin) and agonist (nabilone) of type 1 cannabinoid receptor (CB1R), were administered systemically or locally (into PrL). Chemogenetics, combined Cre-loxP and Flp-FRT system, were employed in mutant mice for neuronal specificity and circuit specificity observation.

**Results:**

After AS, the composite Z score significantly increased at 6 and 9 but not at 24 h, whereas blockade of CB1R systemically and intra-PrL could specifically decrease the composite Z score at 6 and 9 h after AS. Results of fiber photometry further confirmed that the activity of eCB in the PrL was enhanced by AS, especially in the Y maze test at 6 h post-operatively. Moreover, the activation of glutamatergic neurons in the PrL could reduce the composite Z score, which could be significantly reversed by exogenous cannabinoid (nabilone) at 6 and 9 h post-operatively. However, activation of GABAergic neurons only decreased composite Z score at 9 h post-operatively, with no response to nabilone application. Further study revealed the glutamatergic projection from mediodorsal thalamus (MD) to PrL glutamatergic neurons, but not hippocampus (HIP)-PrL circuit, was in charge of the effect of eCBs on POD.

**Conclusion:**

Our study firstly demonstrated the involvement of eCBs in the POD pathogenesis and further revealed that the eCBs may regulate POD through the specific MD^glu^-PrL^glu^ circuit. These findings not only partly revealed the molecular and circuit mechanisms of POD, but also provided an applicable candidate for the clinical prevention and treatment of POD.

## Introduction

With the gradual legalization of cannabis, about 10–20% of the adult population consumes cannabis every year, including 2–3% of the population authorized with medical cannabis (MMP, 2022). Furthermore, it was estimated that 5% of surgical patients might take cannabis in the USA (Davidson et al., 2020). Given the significant psychiatric effects of cannabinoids *via* the type 1 cannabinoid receptor (CB1R), cannabis and its target, the endocannabinoid system (eCBs), may influence post-operative consciousness and cognition (Busquets-Garcia et al., 2015).

Activation of CB1R by cannabis consumption may induce delirium-like behaviors, including delusions, hallucinations, paranoid ideas, impaired memory, and reduced attention spans (Hollister, 1988; Andre et al., 2006; Mack et al., 2012; Kokalj et al., 2016). Furthermore, we previously revealed that general anesthesia may activate eCBs, and blockade of CB1R could accelerate consciousness recovery after general anesthesia (Zhong et al., 2017), indicating eCBs may be associated with delirium after anesthesia and surgery (AS), namely post-operative delirium (POD). As one of the most common perioperative complications, POD affects up to 73.5% of surgical patients (Cui et al., 2020), leading to prolonged hospitalization, 3-fold higher mortality, long-term cognitive decline even dementia, and increased medical costs (Rudolph and Marcantonio, 2011; Daiello et al., 2019; Shi et al., 2019; Labaste et al., 2020). However, the pathogenesis of POD, especially the effect of eCBs on POD, remains largely unclear.

The prefrontal cortex (PFC), a high-order cortex, which plays a vital role in consciousness and cognitive control, is involved in the eCBs associated with recovery from anesthesia (Miller, 2000; Miller and Cohen, 2001; Zhong et al., 2017). Furthermore, the prelimbic prefrontal cortex (PrL), the important part of ventromedial PFC, has been attracting more and more attention in cognition, recognition, learning, and memory (Brockett et al., 2022; Cardoso-Cruz et al., 2022; Garcia-Font et al., 2022). Recent research also showed that increased pro-inflammatory cytokines in the PFC induced by lipopolysaccharide (LPS) led to delirium-like behavior in mice (Zhang et al., 2021). Furthermore, AS decreased excitatory synaptic transmission in PFC pyramidal neurons, but did not change the firing properties of the PFC pyramidal neurons (Matsumoto et al., 2021). Considering the classic retrograde inhibitory effect of CB1R on presynaptic transmitter release, eCBs activated by AS may inhibit the pre-synaptic functions of PFC, resulting in POD.

To verify the hypothesis, especially the POD-associated circuit modulated by the eCBs, we applied chemogenetics, fiber photometry, and behavioral tests. We specifically focused on the PrL (Anastasiades and Carter, 2021), and its upstream brain regions, the mediodorsal thalamus (MD) and hippocampus (HIP). Because the MD-PrL circuit was involved in a wide range of cognitive functions, including working memory, learning, attention, and arousal (Parnaudeau et al., 2018; Sun et al., 2019; Anastasiades et al., 2021), whereas the HIP-PrL circuit plays an essential role in memory encoding and emotion control (Spellman et al., 2015; Sánchez-Bellot and Macaskill, 2020).

In the present study, we confirmed that AS could overexcite the eCB signal in the PrL, which promoted POD through both plasma membrane CB1R (pmCB1R) and mitochondrial CB1R (mtCB1R). Furthermore, the glutamatergic neurons in the PrL innervated by excitatory MD neurons, rather than the HIP-PrL circuit, participated in eCBs-associated POD development. Exogenous cannabinoid nabilone could reverse the protective effect of MD^glu^-PrL^glu^ circuit activation. Our results, for the first time, revealed that cannabinoids play a crucial role in POD pathogenesis, and further clarified the neuronal specificity and circuit specificity of eCBs associated with POD development. These findings partly revealed the molecular and circuit mechanisms of POD and provided a new ideal target for POD prevention and treatment.

## Materials and methods

### Animals

The experimental protocols were approved by the Ethics Committee for Animal Experimentation of the Fourth Military Medical University, and all the experiments were conducted according to the Guidelines for Animal Experimentation of the Fourth Military Medical University (Xi'an, China).

Male C57BL/6 mice, aged 8–12 weeks, were provided by the Experimental Animal Center of the Fourth Military Medical University. Vglut2-Cre mice and Vgat-Cre mice were originated from the Jackson Laboratory. All mice were housed under a 12-h light/dark cycle (lights on from 7:00 to 19:00), 23 ± 1°C temperature, 38–42% humidity, and free access to water and food.

### Stereotactic surgeries

Mice were fixed in the stereotaxic frame (RWD, China) under sodium pentobarbital (50 mg/kg) with erythromycin ophthalmic ointment applied for eye protection. After shaving and skin antisepsis, the scalp was sagittal cut. A heating mat was used to keep the mice warm.

For the chemogenetic experiments, 200 nl/side of AAV-DIO-hM3Dq-mCherry (Brain-VTA, China) (hM3Dq) or AAV-DIO-mCherry (Brain-VTA, China) (control) was microinjected into the bilateral PrL [anteroposterior (AP), +1.98 mm; mediolateral (ML), ±0.4 mm; dorsoventral (DV), −1.8 mm], MD (AP, −1.2 mm; ML, ±0.27 mm; DV, −2.75 mm) or HIP (AP, −2.0 mm; ML, ±1.2 mm; DV, −1.5 mm) at a rate of 50 nl/min in Vglut2-Cre or Vgat-Cre mice. For the experiments using combined Cre-loxP with Flp-FRT system, the AAV-DIO-EGFP-FLPO (OBiO, China) was microinjected into the MD, while the AAV-fDIO-hM3Dq-mCherry (OBiO, China) or AAV-fDIO-mCherry (OBiO, China) was administrated into PrL. The injecting glass electrode was left for 10 min and then slowly retrieved.

For fiber photometry, 300 nl of AAV-hsyn-eCB2.0 (WZ Bioscience Inc., China) was microinjected into PrL followed by the optical fiber [2.5 mm in diameter, 200 μm fiber optic cable, numerical aperture (NA) = 0.37, Inper, China] insertion into the PrL.

The dual guide cannulas (0.21 mm in diameter, RWD, China) were inserted into the PrL of mice for pharmacological administration.

Optical fibers and guide cannulas were fixed using methyl methacrylate cement. Animals were allowed to recover for at least 3 weeks after the virus injection, or 5–7 days after cannula implantation before further studies.

### POD model establishment and pharmacological intervention

As previously described (Peng et al., 2016), mice in AS group underwent a simple laparotomy under isoflurane anesthesia (1.4% isoflurane delivered with 100% O_2_ at 1.0 L/min), whereas mice in the sham group were only placed in the anesthesia chamber with room air without any surgery. The total inhalation time was 2 h in both groups. During the simple laparotomy, the midline abdominal skin, muscles, and peritoneum were longitudinally dissected layer by layer, from the xiphoid to 0.5 cm above the pubic symphysis. An ~3-cm long segment of the small intestine was exposed outside the abdominal cavity for 2 min. After putting the small intestine back to place, the incision was sutured layer by layer with 5–0 Vicryl. Then, EMLA cream (2.5% lidocaine and 2.5% prilocaine) was applied on the incision wound and then every 8 h for 1 day.

In the pharmacological studies, all drugs, dissolved in a solvent mixture of dimethylsulfoxide (DMSO), Tween 80, and saline (1:1:18), were administrated to mice 15 min before the anesthesia. The selective CB1R antagonists, AM281 and hemopressin (Hemo), were administrated intraperitoneally at a dose of 3 and 1 mg/kg, respectively; or microinjected into the PrL at a dose of 100 ng/200 nl/side. Therefore, AM281 could block CB1R on both the cell membrane and mitochondrial membrane, while the Hemo could only block the plasma membrane CB1R (pmCB1R) (Benard et al., 2012). The synthetic cannabinoid, nabilone, was administrated intraperitoneally at a dose of 1 mg/kg or microinjected into the PrL at a dose of 100 ng/200 nl/side through an injector cannula. All of these agents were obtained from Sigma-Aldrich (USA).

### Behavioral tests

The behavioral tests were performed as described in previous studies with modifications (Peng et al., 2016). All mice had multiple behavioral tests in the order of buried food test, open field test (OFT), and Y maze test (YMT) at 24 h before (baseline) the AS and at 6, 9, and 24 h after the AS. All tests were conducted in a quiet and illuminated room. The mice were habituated in the testing room for 1 h before the test. The apparatus was cleaned with 70% ethanol and dried between each test.

#### Buried food test

In the test cage with 3-cm-high clean bedding, one sweetened cereal was buried 0.5 cm below the surface of the bedding at a random location. The mouse was placed softly in the center of the test cage and allowed to explore for 5 min. The latency was recorded as the duration from the mouse was located in the test cage to its grasped food pellet in the forepaws and/or teeth. If the mouse failed to find the cereal within 5 min, the latency was defined as 300 s.

#### Open field test

The mouse was gently placed in the corner of a square chamber (40 × 40 × 40 cm) and allowed to move freely for 5 min. The movement was recorded and the total distance moved, time spent in the center, the freezing time, and the latency to the center were analyzed by the Any-Maze animal tracking system software (Stoelting Co., USA).

#### Y maze test

The standard Y maze used in the study includes the start arm, novel arm, and other arm (8 × 30 × 15 cm) with an angle of 120 degrees between each arm. In the first trial, the mouse was placed in the start arm and allowed to explore the start arm and other arm for 10 min, with the novel arm being blocked. After a 1-h inter-trial interval (ITI), the second trial (retention) was conducted, during which the mouse was placed back in the start arm with free access to all three arms for 5 min. The behavior of mice was monitored, and the number of entries in each arm and the time spent in the novel arm were analyzed by the Any-Maze animal tracking system software.

### Chemogenetic experiments

To manipulate the glutamatergic and GABAergic neurons in the PrL, clozapine N-oxide (CNO) (Sigma-Aldrich, USA) at a dose of 1 mg/kg or an equivalent volume of saline was intraperitoneally injected. To explore the function of glutamatergic terminals in the PrL from MD or HIP, 5 μM CNO or saline was microinjected intra the PrL at 0.06 μL/min for 5 min. Thirty min after CNO administration, the anesthesia and surgery were conducted.

### Fiber photometry recording

At least 14 days after the viral administration and optic fiber implantation, photometry recording was performed using the commercial photometry system (Thinker Tech, China) in mice during the AS and behavioral tests. The 470 nm LED was used to excite eCB sensors, and the 405 nm LED was used to obtain the isosbestic signal. The photometry data were recorded and exported to MATLAB for further analysis. Account for photobleaching, the raw signals were first adjusted (Dong et al., 2021). For each trial, the averaged baseline signals before stimuli presentation were considered as “F0” and the change of fluorescence (ΔF/F) was calculated as (F–F0)/F0.

### Immunohistochemistry

Mice were deep anesthetized and transcardially perfused with 0.9% saline followed by 4% paraformaldehyde (PFA). Brains were post-fixed for 2 h in 4% PFA at 4°C and then dehydrated by 30% sucrose. Subsequently, brains were coronally sectioned at 40 μm using a cryostat microtome (CM1200, Leica, Germany). The sections were washed in phosphate-buffered saline (PBS, pH = 7.4) and then blocked with 5% normal donkey serum in PBS with 0.3% Triton X-100 (PBST) for 2 h at room temperature. Then, the sections were incubated in 2.5% donkey serum with PBST for 24 h at 4°C with guinea pig anti-c-Fos antibody (1:1,000, 226308, Synaptic Systems, Germany), rabbit anti-glutamate antibody (1:500, G6642, Sigma-Aldrich, USA), and rabbit anti-GABA antibody (1:200, GTX125988, GeneTex, USA), respectively. Afterwards, secondary antibodies, including donkey anti-guinea pig Alexa Fluor 488 (1:500, 706-545-148, Jackson ImmunoResearch, USA), donkey anti-guinea pig Alexa Fluor 594 (1:500, 706-585-148, Jackson ImmunoResearch, USA), donkey anti-guinea pig Alexa Fluor 647 (1:500, 706-605-148, Jackson ImmunoResearch, USA), and donkey anti-rabbit Alexa Fluor 488 (1:500, 711-545-152, Jackson ImmunoResearch, USA), were applied for 2 h at room temperature. At last, the slices were washed and mounted in Fluoromount-G (Millipore, USA) and imaged by laser confocal microscopes (FV1200, Olympus, Japan).

### Western blot analysis

Mice were decapitated under deep anesthesia, and the PrL was rapidly separated and frozen at −80°C until use. The protein was abstracted using lysate. Protein concentration was estimated using a bicinchoninic acid (BCA) kit (Beyotime, China), and 30 μg samples were loaded onto polyacrylamide-SDS gels. Gels were electrophoresed and then transferred to PVDF membranes. Membranes were blocked with a blocking buffer containing 3% bovine serum albumin (Sigma-Aldrich, USA) for 60 min and probed with antibodies directed toward CNR-1 (1:1,000, Santa Cruz, USA) or GAPDH (1:2,000, ABclonal, China) overnight at 4°C. Primary antibody staining was detected with horseradish peroxidase-conjugated goat anti-rabbit antibodies (1:10,000, Beyotime, China). Each group was immune-blotted in three independent experiments, and average optical density relative to the internal standard (GAPDH) was reported and analyzed (Bio-Rad, USA).

### Statistics

All data were expressed as mean ± standard error of the mean (SEM) and analyzed by the Prism 6 software (GraphPad Software, CA). One-way ANOVA followed by Bonferroni's multiple comparisons was used to assess the differences between groups. Two-way ANOVA followed by Bonferroni's multiple comparisons was applied for photometry analysis. The immunohistochemical stainings were analyzed by unpaired *t*-test. *P* < 0.05 were considered statistically significant.

All the behavior parameters were presented as a percentage of those of the baseline for the same group. The POD-like behavioral impairment was shown by composite Z score (Peng et al., 2016), which was calculated as the sum of Z scores of six behavioral tests normalized with the SD for that sum in the shams, including latency to eat food, time spent in the center, latency to the center, freezing time, entries in the novel arm, and duration in the novel arm. Each Z score of the behavioral parameter was calculated as Z = [ΔX_treatment_-MEAN(ΔX)_sham_]/SD(ΔX)_sham_. In the formula, ΔX_sham_ represents the score differences between after and before sham management in the sham group; ΔX_treatment_ was the value that the score of post-treatment, including AS and pharmacological interventions, minus baseline; MEAN(ΔX)_sham_ and SD(ΔX)_sham_ were the mean and the standard deviation of ΔX_sham_, respectively. The impairment of cognition was indicated by the reduction, rather than increase, of time spent in the center, freezing time, entries in the novel arm, and duration in the novel arm. The above four parameters were multiplied by −1 before the composite Z score calculation.

## Results

### Blockade of global CB1R alleviated POD

**Figure 1:**
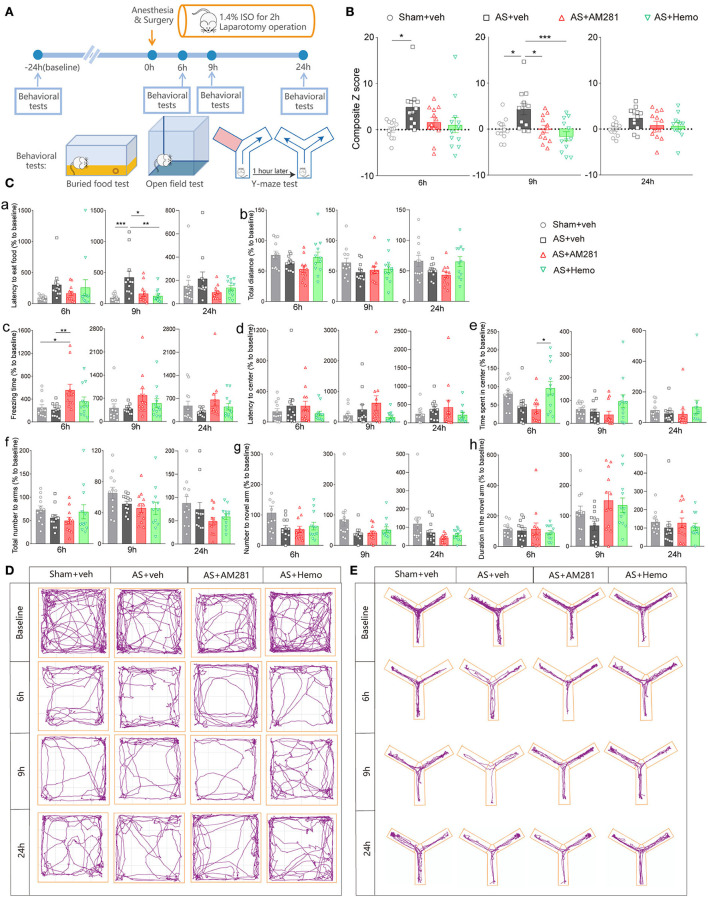
Blockade of global CB1R alleviated POD. **(A)** Scheme of the experimental design. **(B)** The composite Z score at 6 h (left), 9 h (middle), and 24 h (right) after AS. **(C)** The latency to eat food in buried food test (a); the total distance (b), the freezing time (c), the latency to center (d), time spent in center (e) in the open field; the total number to arm (f), number to novel arm (g), duration in the novel arm (h) in Y maze at 6 h (left), 9 h (middle), and 24 h (right). **(D,E)** Running tracks of the mice in an open field **(D)** and Y maze **(E)** in groups. Data are shown as mean ± SEM. *N* = 12. *P < 0.05, **P < 0.01, ***P < 0.001. ISO, isoflurane; AS, anesthesia and surgery; Hemo, hemopressin.

Firstly, we investigated the involvement of eCBs in POD using the AS model and composite Z score according to the previous studies (Peng et al., 2016; Figure 1A). As shown in Figure 1B, the composite Z score was significantly elevated after AS at both 6 h [*F*_(3, 44)_ = 3.010, *P* = 0.0401; Figure 1B left] and 9 h [*F*_(3, 44)_ = 6.850, *P* = 0.0007; Figure 1B middle], indicating the POD model was successfully established. However, the composite Z score was not changed at 24 h after AS [*F*_(3, 44)_ = 2.116, *P* = 0.1118; Figure 1B right]. Therefore, we only investigated behaviors at 6 and 9 h in the further investigation.

Moreover, systemic application of CB1R selective antagonist, AM281 (3 mg/kg) and Hemo (1 mg/kg) could partly reverse the increased composite Z score induced by AS, particularly at 9 h post-operatively with statistical differences (Figure 1B). Both AM281 and Hemo could reverse the prolonged latency to food after AS [*F*_(3, 44)_ = 7.263, *P* = 0.0005; Figure 1Ca]. The freezing time in OFT significantly increased in the AM281 group compared to the sham and AS groups at 6 h after AS [*F*_(3, 44)_ = 4.677, *P* = 0.0064; Figure 1Cc]. The above results confirmed that the eCB signaling was involved in the POD development. The other parameters shown no significant differences (Figures 1Cb,d–h). The running tracks of the mice in open field and Y maze in groups as shown in Figures 1D,E, respectively.

### The PrL was involved in the eCB-associated POD regulation

Then, the involvement of the PFC in the eCB-associated POD regulation was examined by c-Fos staining. Interestingly, the expression of c-Fos in PrL was significantly decreased at 6 and 9 h after AS, which could be reversed by AM281 administration at 9 h after AS (Supplementary Figure 1). On the contrary, the expression of c-Fos in IL (infralimbic cortex) was decreased after AS, but could not be rescued by AM281. Therefore, the PrL was selected in the further experiment.

**Figure 2:**
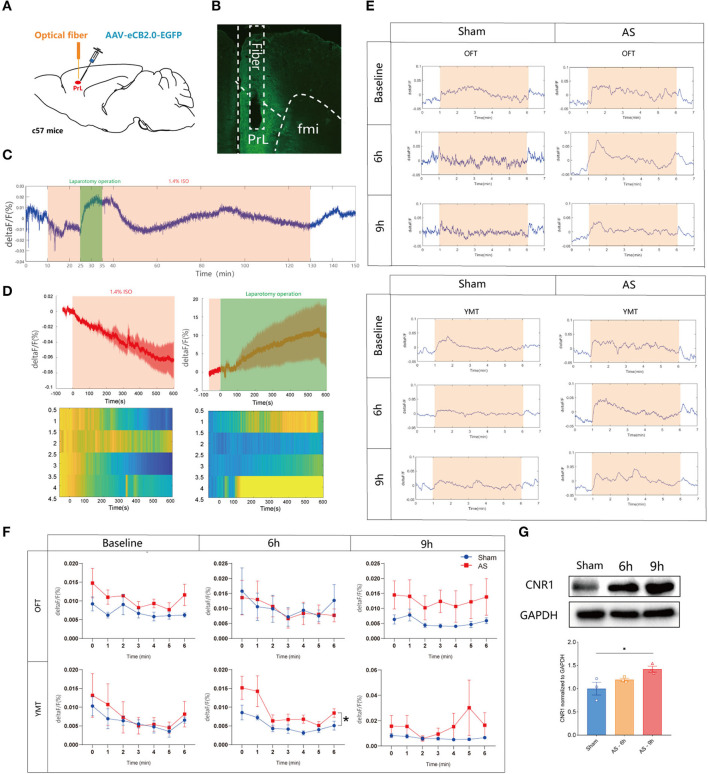
The eCB signals in the PrL during AS and behavioral tests. **(A)** Scheme of the viral microinjection and fiber implantation. **(B)** The fiber location and viral expression. **(C,D)** The typical traces **(C)** and analyzed figure **(D)** of the eCB signal during AS. **(E,F)** The typical curves **(E)** and statistical chart **(F)** of the eCB signal during the open field and Y maze tests. **(G)** The expression of CB1R in the PrL after AS. Data are shown as mean ± SEM. *N* = 4. *P < 0.05. AS, anesthesia and surgery; OFT, open field test; YMT, Y maze test.

To reveal the eCB dynamics in PrL before and after AS, a fluorescent sensor for spatiotemporal detection was employed. Two weeks after the microinjection of the eCB sensor intra the PrL and optical fibers implantation (Figure 2A), the eCB signaling was monitored during AS and the behavioral tests, and the expression of the sensor virus was verified (Figure 2B). We found that the eCB signal of PrL was gradually weakened after the initiation of isoflurane anesthesia and boosted during the short operation (Figures 2C,D). Afterward, the signal kept fluctuating until the cessation of anesthesia and gradually recovered to the baseline level at 20 min after anesthesia. However, the eCB signals exhibited an increasing tendency during the behavioral tests mostly without significance. In the YMT, the ΔF/F value of eCB signaling in AS group was significantly higher than in the sham group [*F*_(1, 6)_ = 7.184, *P* = 0.0365; Figures 2E,F] at 6 h, but not 9 h after AS [*F*_(1, 6)_ = 1.372, *P* = 0.2859]. Besides, the expression of the CB1R in the PrL was time-dependently increased after AS, especially at 9 h post-operative (1.000 ± 0.1364 vs. 1.419 ± 0.06338, *P* = 0.0360; Figure 2G).

**Figure 3:**
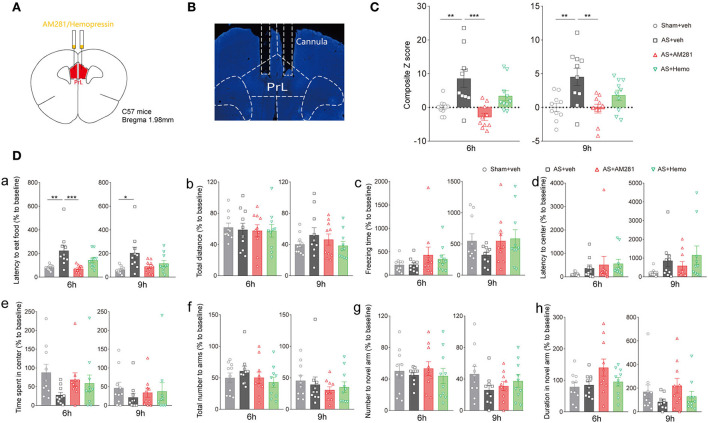
Blockade of CB1R in the PrL promoted the composite Z score. **(A)** Scheme of the drug administration. **(B)** The site of the cannula in the PrL. **(C)** The composite Z score at 6 h (left) and 9 h (right) after AS. **(D)** The latency to eat food in buried food test (a); the total distance (b), the freezing time (c), the latency to center (d), time spent in center (e) in the open field; the total number to arm (f), number to novel arm (g), duration in the novel arm (h) in Y maze at 6 h (left) and 9 h (right) after AS. Data are shown as mean ± SEM. *N* = 10. *P < 0.05, **P < 0.01, ***P < 0.001. ISO, isoflurane; AS, anesthesia and surgery; Hemo, hemopressin.

Next, we bilateral microinjected AM281 (100 ng/200 nl/side) or Hemo (100 ng/200 nl/side) intra the PrL 15 min before AS to verify the role of the PrL CB1R in POD pathogenesis (Figure 3A). The location of the cannulas was confirmed after behavioral tests (Figure 3B). We found that the composite Z score in the AM281 group, but not in the Hemo group, was significantly lower than in the AS group at 6 h [*F*_(3, 36)_ = 8.869, *P* = 0.0002] and 9 h [*F*_(3, 6)_ = 6.405, *P* = 0.0014] post-operatively (Figure 3C), indicating both plasma and mitochondrial CB1R were important in POD regulation. Specifically, the latency to food at 6 h was prolonged in AS group, which was reversed by AM281 [*F*_(3, 36)_ = 7.437, *P* = 0.0005; Figure 3Da]. The other parameters shown no significant differences (Figures 3Db–h). These findings indicated that the eCB signaling in the PrL was associated with POD, presumably through the increased endocannabinoids and CB1R (Riederer et al., 2016).

### eCB signaling modulated POD through the glutamatergic neurons rather than GABAergic neurons in the PrL

**Figure 4:**
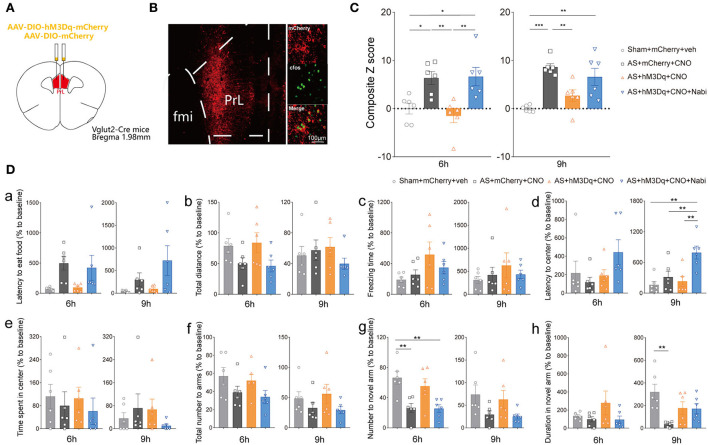
eCB signaling modulated POD through the PrL glutamatergic neurons. **(A)** Scheme of hM3Dq or control virus injection into the PrL of Vglut2-Cre mice. **(B)** The injection site (left) and activation efficiency (right). **(C)** The composite Z score at 6 h (left) and 9 h (right) after AS. **(D)** The latency to eat food in buried food test (a); the total distance (b), the freezing time (c), the latency to center (d), time spent in center (e) in the open field; the total number to arm (f), number to novel arm (g), duration in the novel arm (h) in Y maze at 6 h (left) and 9 h (right) after AS. Data are shown as mean ± SEM. *N* = 6. *P < 0.05, **P < 0.01, ***P < 0.001. ISO, isoflurane; AS, anesthesia and surgery; Nabi, nabilone; CNO, clozapine N-oxide; mCherry, AAV-DIO-mCherry; hM3Dq, AAV-DIO-hM3Dq-mCherry.

To clarify the neuronal specificity of PrL in POD regulation, we further applied chemogenetics and pharmacological interventions in Vglut2-Cre mice and Vgat-Cre mice (Figure 4A, Supplementary Figure 2A). After 3 weeks of viral expression, the mice were applied with AS followed by behavioral tests. The expression and functions of AAV-DIO-hM3Dq-mCherry (hM3Dq) and AAV-DIO-mCherry (control) viruses were verified, as evidenced by Figure 4B and Supplementary Figure 2B.

We found that activation of PrL glutamatergic neurons in the AS + hM3Dq + CNO group significantly reduced the composite Z score compared with the control (AS + mCherry + CNO) group at both 6 h [*F*_(3, 20)_ = 8.079, *P* = 0.0010] and 9 h [*F*_(3, 20)_ = 11.50, *P* = 0.0001] post-operatively (Figure 4C). However, activation of PrL GABAergic neurons could only relieve the behavioral impairment at 9 h after AS [*F*_(3, 20)_ = 10.99, *P* = 0.0007; Supplementary Figure 2C].

Furthermore, activation of CB1R by nabilone (intraperitoneally at a dose of 3 mg/kg) could only reverse the POD-preventing effect induced by activating PrL glutamatergic neurons (Figure 4C) at 6 h, but not by activation of GABAergic neurons at 6 h [*F*_(3, 20)_ = 3.356, *P* = 0.0393; Supplementary Figure 2C]. Nabilone is an exogenous cannabinoid that exerts CB1R-activating effects similar to cannabis consumption. Compared with the sham group, AS and nabilone administration with the activation of glutamatergic neurons significantly reduced the numbers to the novel arm in YMT at 6 h [*F*_(3, 20)_ = 7.452, *P* = 0.0015; Figure 4Dg]. Notably, the nabilone application exhibited significantly longer latency to center in OFT than the other three groups at 9 h post-operatively [*F*_(3, 20)_ = 7.754, *P* = 0.0013; Figure 4Dd], suggesting an enhanced anxiety level induced by nabilone after AS. The other parameters shown no significant differences (Figures 4Da–c,e,f,h). Above findings indicated the PrL glutamatergic neurons, rather than GABAergic neurons, might play an essential role in the eCBs mediated POD regulation.

### The MD^glu^-PrL^glu^ circuit, but not the HIP^glu^-PrL circuit, was responsible for the effect of eCB on POD

Given MD-PrL (Parnaudeau et al., 2018) and HIP-PrL (Sánchez-Bellot and Macaskill, 2020) were involved in the regulation of working memory and long-term memory, respectively, we investigated the roles of the two circuits in POD modulation in the further study. The retrograde viruses were used to label the upstream neurons of the PrL glutamatergic neurons in MD and HIP (Supplementary Figure 3A). Two weeks after being injected with the helper viruses (AAV-DIO-H2B-EGFP-T2A-TVA and AAV-DIO-oRVG) and RV-ENVA-NVAdsRed (RV), we co-labeled the glutamatergic and GABAergic neurons in MD and HIP.

Co-expression of the RV and helper viruses in the PrL was verified (Supplementary Figure 3B). As shown in Supplementary Figure 3C, the PrL glutamatergic neurons were innervated by MD glutamatergic neurons more than MD GABAergic neurons (0.47 ± 0.06 vs. 0.29 ± 0.07, *P* = 0.0476; Supplementary Figures 3C,D). However, there were no significant retrograde RV-labeled neurons in the HIP (Supplementary Figure 3E).

**Figure 5:**
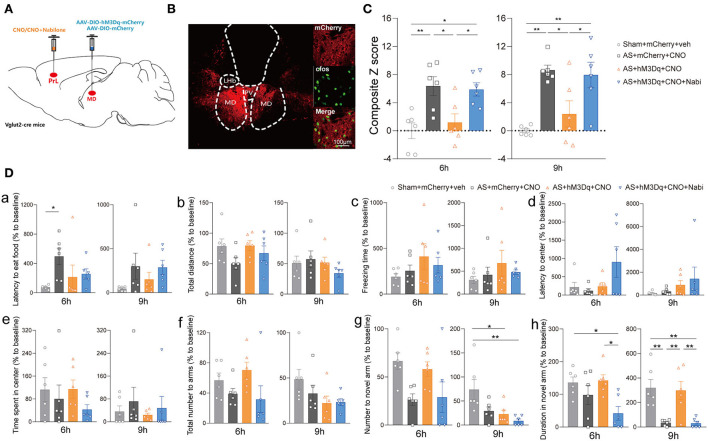
The MD^glu^-PrL circuit was responsible for the effect of eCBs on POD. **(A)** Scheme of viral injection and CNO application. **(B)** The injection site (left) and activation efficiency (right). **(C)** The composite Z score at 6 h (left) and 9 h (right) after AS. **(D)** The latency to eat food in buried food test (a); the total distance (b), the freezing time (c), the latency to center (d), time spent in center (e) in the open field; the total number to arm (f), number to novel arm (g), duration in the novel arm (h) in Y maze at 6 h (left) and 9 h (right) after AS. Data are shown as mean ± SEM. *N* = 6. *P < 0.05, **P < 0.01. AS, anesthesia and surgery; Nabi, nabilone; CNO, clozapine N-oxide; mCherry, AAV-DIO-mCherry; hM3Dq, AAV-DIO-hM3Dq-mCherry; PrL, prelimbic cortex; MD, mediodorsal thalamus.

Furthermore, we modulated MD^glu^-PrL and HIP^glu^-PrL circuits to investigate their roles in POD pathogenesis in Vglut2-Cre mice (Figures 5A,B). Activation of MD^glu^-PrL could statistically alleviate POD at 6 h [*F*_(3, 20)_ = 7.606, *P* = 0.0014] and 9 h [*F*_(3, 20)_ = 9.646, *P* = 0.0004] compared with AS+mCherry+CNO group (Figure 5C). Meanwhile, nabilone administration (100 ng/200 nl/side) intra the PrL could partly reverse this relieving effect at 6 and 9 h compared with the AS + hM3Dq + CNO group (Figure 5C). It is worth noting that the duration stays in the novel arm in YMT was greatly improved by activation of MD^glu^-PrL compared to the AS group at 9 h after AS [*F*_(3, 20)_ = 9.691, *P* = 0.0004; Figure 5Dh]. The other parameters shown no significant differences (Figures 5Da–g). However, activation of HIP^glu^-PrL failed to rescue POD [*F*_(3, 20)_ = 2.965, *P* = 0.0567 at 6 h; *F*_(3, 20)_ = 3.664, *P* = 0.0297 at 9 h; Supplementary Figure 4].

**Figure 6:**
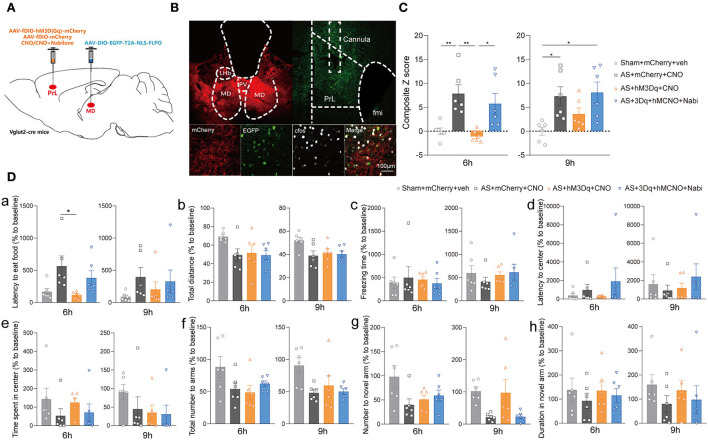
The MD^glu^-PrL^glu^ circuit was involved in the effect of eCB on POD. **(A)** Scheme of viral injection and CNO application. **(B)** The injection site in MD and PrL (above) and activation efficiency (below). **(C)** The composite Z score at 6 h (left) and 9 h (right) after AS. **(D)** The latency to eat food in buried food test (a); the total distance (b), the freezing time (c), the latency to center (d), time spent in center (e) in the open field; the total number to arm (f), number to novel arm (g), duration in the novel arm (h) in Y maze at 6 h (left) and 9 h (right) after AS. Data are shown as mean ± SEM. *N* = 6. *P < 0.05, **P < 0.01. AS, anesthesia and surgery; Nabi, nabilone; CNO, clozapine N-oxide; mCherry, AAV-fDIO-mCherry; hM3Dq, AAV-fDIO-hM3Dq-mCherry; PrL, prelimbic cortex; MD, mediodorsal thalamus.

Next, we investigated the effect of the specific MD^glu^-PrL^glu^ circuit on eCB signaling-related POD regulation using the combined Cre-loxP and Flp-FRT system in Vglut2-Cre mice. Briefly, the AAV-DIO-EGFP-FLPO was microinjected into the MD, while the AAV-fDIO-hM3Dq or AAV-fDIO-mCherry was microinjected into the PrL (Figures 6A,B). Selective activation of the MD^glu^-PrL^glu^ circuit could decrease the composite Z score, particularly 6 h after AS compared with the AS + mCherry + CNO group while nabilone administration (100 ng/200 nl/side) intra the PrL reversed the protective effect [*F*_(3, 20)_ = 8.470, *P* = 0.0008; Figure 6C]. In the buried food test, the latency to eat food in the AS + mCherry + CNO group was significantly more extended than in the AS + hM3Dq + CNO group [*F*_(3, 20)_ = 4.126, *P* = 0.0198; Figure 6Da] 6 h after AS. The other parameters shown no significant differences (Figures 6Db–h). These findings further confirmed that the MD^glu^-PrL^glu^ circuit was responsible for the effect of eCB on POD.

## Discussion

In this study, we demonstrated the role and underlying circuit mechanisms of eCB signaling in POD regulation for the first time. We found that blockade of CB1R in the PrL could effectively alleviate POD through the glutamatergic neurons rather than GABAergic neurons. Furthermore, the post-operative eCB signaling, activated by AS, may regulate the function of the MD^glu^-PrL^glu^ circuit, but not HIP^glu^-PrL projection. Exogenous cannabinoids aggravate POD, which was alleviated by the activation of MD-PrL. Our results suggest the increased eCB signal in the PrL may be a critical factor for POD pathogenesis, which may also be involved in the mechanisms of marijuana smoking-associated POD.

In the present study, we used two antagonists of CB1R: AM281, the classical selective reversal antagonist of CB1R, is lipid-soluble and able to block CB1R on both cell membrane and mitochondrial membrane; while the Hemo could only block the plasma membrane CB1R (pmCB1R) because it is unable to penetrate plasma membranes (Benard et al., 2012). As expected, AM281 showed a significant alleviating effect on POD rather than Hemo, indicating both the pmCB1R and mitochondrial CB1R (mtCB1R) are necessary for the regulating effect of eCBs on POD. A series of studies have shown that mtCB1R also played an essential role in synaptic function in cognitive and emotional modulation (Cardinal et al., 2012; Djeungoue-Petga and Hebert-Chatelain, 2017). Activation of mtCB1R could decrease mitochondrial ATP production, calcium buffering capacity, ROS generation, and the mobility of neuronal mitochondria to inhibit transmitter release in the synapses (Cardinal et al., 2012; Djeungoue-Petga and Hebert-Chatelain, 2017). Therefore, eCBs may modulate the MD-PrL circuit by the pmCB1R-associated classical retrograde synaptic inhibition (Busquets-Garcia et al., 2015) and mtCB1R-related presynaptic metabolism inhibition.

Within the PrL, we found activation of both glutamatergic and GABAergic neurons could alleviate the POD behavior. However, the influence of PrL glutamatergic neuronal activation was earlier and more prominently than GABAergic neurons, suggesting a temporal sequence for PrL neurons' recovery from AS. The imbalance of PrL glutamatergic and GABAergic neurons recovery could significantly affect the function of the higher cortex, including mood, memory, and states of consciousness (Lacreuse et al., 2018; Dienel et al., 2020; Fogaca et al., 2021; Quinones et al., 2021; Sabihi et al., 2021; Smucny et al., 2021; Yan and Rein, 2021), which may lead to POD. However, activation of PrL GABAergic neurons seemed that they did not further inhibit the local glutamatergic neurons to aggravate cognitive impairments, but alleviated POD probably through other long-range projections as in HIP and amygdala (Alkire et al., 2008; Sun et al., 2019; Guo et al., 2021; Shepherd and Yamawaki, 2021). On the other hand, exogenous cannabinoid only reversed the cognitive relieving effect of glutamatergic neurons, rather than GABAergic neurons, indicating the influence of CB1R on POD in the PrL exhibited cellular specificity. The phenomenon may be attributed to the cardinal effect of glutamatergic neurons in primary information processing, conducting, and amplifying (Sherman, 2016). Otherwise, cannabinoid may selectively act on the pre-synaptic CB1R of glutamatergic neurons in the PrL.

To examine the above hypotheses, we retrogradely traced the PrL glutamatergic neurons and found that MD, instead of the HIP, is the most important upstream nucleus of PrL. PrL was first defined as the cortex that receives projections from the thalamus, further, MD shares rich reciprocal connections with PrL (Parnaudeau et al., 2013, 2018; Ahrlund-Richter et al., 2019; Lyuboslavsky et al., 2021). However, no RV-labeled neurons were found in the HIP, which may be due to the regional specificity of HIP inputs to the PrL, including strong inputs from multiple layers in the intralimbic area (IL) and weak restricted input from Layer 5 in PrL. Combined with the efficiency and sparse diffusion of viruses, which were predominantly microinjected in Layer 3 in our experiment, the negative result was reasonable (Liu and Carter, 2018). Although the thalamus was believed to be the relay station for integrating various cognitive functions (Wen et al., 2021; Wolff et al., 2021), more and more pieces of evidence confirmed that the thalamus is also engaged in essential and unique computations of cognition through projections to the cortex (Mitchell et al., 2014; Nakajima and Halassa, 2017; Rikhye et al., 2018). The MD projections could sustain PrL activity during working memory maintenance (Bolkan et al., 2017). Inconsistent with the precious results, we confirmed that activation of the MD^glu^-PrL^glu^ circuit could noticeably improve POD, implying the strength of thalamus-cortex connections was impaired by anesthesia and surgery. Thus, the pyramidal neurons in the PrL were de-excited to maintain normal cognition and emotion, resulting in delirium-like behavior. Combined with the results of the eCBs signaling, the impaired excitatory MD-PrL projection was due to the CB1R-related presynaptic inhibition. These results also supported Yu Matsumoto's findings (Matsumoto et al., 2021) that AS decreased the excitatory presynaptic responses of PrL pyramidal neurons without any changes in the response kinetics.

There are two limitations of the present study. Firstly, we only investigated the MD-PrL and HIP-PrL circuit, however, there are abundant afferent and efferent projections of PrL. Future research is needed to explore other upstream projections and the specific downstream targets mediated emotion and consciousness, such as BLA-PrL (Liu et al., 2020) and DMH-PrL (Zhong et al., 2017). Secondly, we did not further probe the function of PrL GABAergic neurons, which also participated in the POD development. Though activation of PrL GABAergic neurons could not reverse the effect of nabilone, we cannot exclude the involvement of the PrL GABAergic neurons in eCB-associated POD.

In summary, our study, for the first time, demonstrated the involvement of eCB in POD pathogenesis and further revealed that eCB signaling activated by anesthesia and surgery and exogenous cannabinoid could promote POD through the MD^glu^-PrL^glu^ circuit. These findings not only expanded the understanding of POD from both molecular and circuit aspects but may also provide new applicable therapeutic targets for POD prevention and treatment in the future.

## Data availability statement

The original contributions presented in the study are included in the article/Supplementary material, further inquiries can be directed to the corresponding author.

## Ethics statement

The animal study was reviewed and approved by Ethics Committee for Animal Experimentation of the Fourth Military Medical University.

## Author contributions

YL designed the study, conducted the study, analyzed the data, and wrote the manuscript. SJ and JW conducted the study. DW, XZ, HL, FZ, ZZ, and QL helped conducted the study and analyzed the data. HZ and HD designed the study and polished the manuscript. All authors contributed to the article and approved the submitted version.

## Funding

This work was supported by the National Natural Science Foundation of China (Grant Nos. 82071487, 81771427 to HZ and Grant No. 82030038 to HD) and the Natural Science Basic Research Program of Shaanxi province (Grant Nos. 2020JM-313, 2022JC-59 to HZ).

## Conflict of interest

The authors declare that the research was conducted in the absence of any commercial or financial relationships that could be construed as a potential conflict of interest.

## Publisher's note

All claims expressed in this article are solely those of the authors and do not necessarily represent those of their affiliated organizations, or those of the publisher, the editors and the reviewers. Any product that may be evaluated in this article, or claim that may be made by its manufacturer, is not guaranteed or endorsed by the publisher.

## Supplementary material

Supplementary Figure 1 The expression of the c-Fos in the PrL after AS. **(A)** The expression of c-Fos (red) in the PrL and IL after AS with vehicle or AM281 administration. **(B)** The changes of c-Fos expression in the PrL. **(C)** The changes of c-Fos expression in the IL. *N* ≥ 3. ^*^*P* < 0.05, ^**^*P* < 0.01, ^***^*P* < 0.001. AS, anesthesia & surgery; PrL, prelimbic cortex.

Supplementary Figure 2 PrL GABAergic neurons modulated POD without responses to cannabinoid. **(A)** Scheme of viral injection. **(B)** The injection site (left) and activation efficiency (right). **(C)** The composite Z score at 6 h (left) and 9 h (right) after AS. **(D)** The latency to eat food in buried food test (a); the total distance (b), the freezing time (c), the latency to center (d), time spent in center (e) in open field; the total number to arm (f), number to novel arm (g), duration in the novel arm (h) in Y maze at 6 h (left) and 9 h (right) after AS. Data are shown as mean ± SEM. *N* = 6. ^*^*P* < 0.05, ^**^*P* < 0.01, ^***^*P* < 0.001. ISO, isoflurane; AS, anesthesia & surgery; Nabi, nabilone; CNO, clozapine N-oxide; mCherry, AAV–DIO-mCherry; hM3Dq, AAV-DIO-hM3Dq-mCherry; PrL, prelimbic cortex.

Supplementary Figure 3 The RV retrograde labeled neurons in the MD and HIP from the PrL glutamatergic neurons. **(A)** Scheme of injection of helper and RV viruses into the PrL of Vglut2-Cre mice. **(B)** The expression of the viruses in the PrL. **(C)** The co-localization of RV-labeled neurons and glutamatergic and GABAergic neurons in the MD. Arrowheads indicate the co-labeled cells. **(D)** The percentage of glutamatergic (blue bar) and GABAergic neurons (red bar) in the RV-labeled neurons. **(E)** The co-localization of RV-labeled neurons and glutamatergic and GABAergic neurons in the HIP. Data are shown as mean ± SEM. *N* ≥ 3. ^*^*P* < 0.05, ^****^*P* < 0.0001. RV, RV-ENVA-ΔG-dsRed; Helper, AAV-DIO-H2B-EGFP-T2A-TVA & AAV-DIO-oRVG; PrL, prelimbic cortex; MD, mediodorsal thalamus.

Supplementary Figure 4 Activation of HIP^glu^ - PrL circuit did not affect POD. **(A)** Scheme of viral injection and drug application. **(B)** The injection site (left) and activation efficiency (right). **(C)** The composite Z score at 6 h (left) and 9 h (right) after AS. **(D)** The latency to eat food in buried food test (a); the total distance (b), the freezing time (c), the latency to center (d), time spent in center (e) in open field; the total number to arm (f), number to novel arm (g), duration in the novel arm (h) in Y maze at 6 h (left) and 9 h (right) after AS. Data are shown as mean ± SEM. *N* = 6. ^*^*P* < 0.05. AS, anesthesia & surgery; Nabi, nabilone; CNO, clozapine N-oxide; mCherry, AAV-DIO-mCherry; hM3Dq, AAV-DIO-hM3Dq-mCherry; PrL, prelimbic cortex; HIP, hippocampus.
