# Supplementary figures and images for: Endocannabinoid signaling regulates post-operative delirium through glutamatergic mediodorsal thalamus-prelimbic prefrontal cortical projection
Source: Front Aging Neurosci. 2022 Dec 1;14:1036428. doi: 10.3389/fnagi.2022.1036428 (PMC9752096; doi:10.3389/fnagi.2022.1036428)

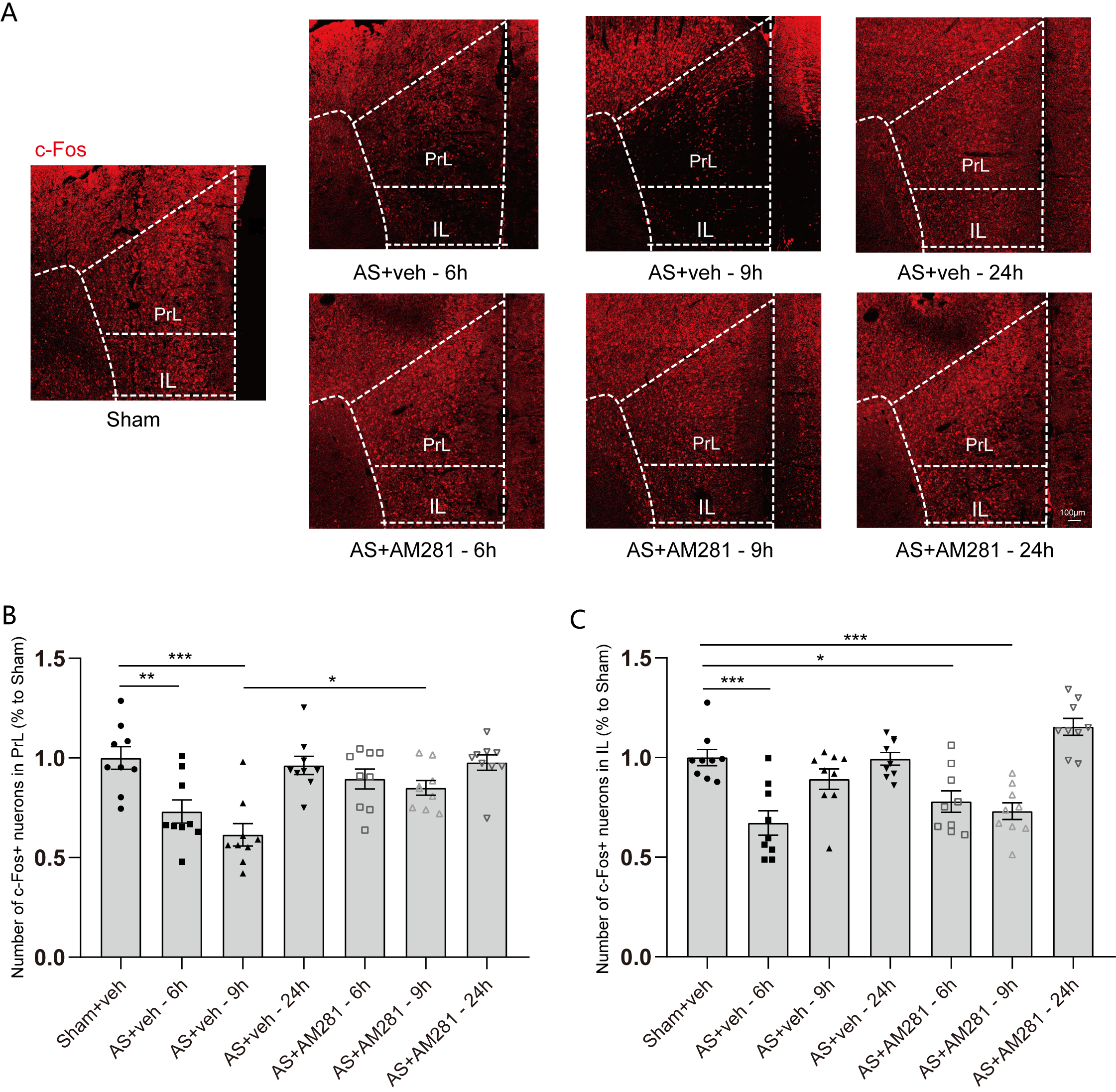

Supplement: Supplementary Figure 1 — The expression of the c-Fos in the PrL after AS. (A) The expression of c-Fos (red) in the PrL and IL after AS with vehicle or AM281 administration. (B) The changes of c-Fos expression in the PrL. (C) The changes of c-Fos expression in the IL. N ≥ 3. *P < 0.05, **P < 0.01, ***P < 0.001. AS, anesthesia & surgery; PrL, prelimbic cortex. [file Image_1.TIF]

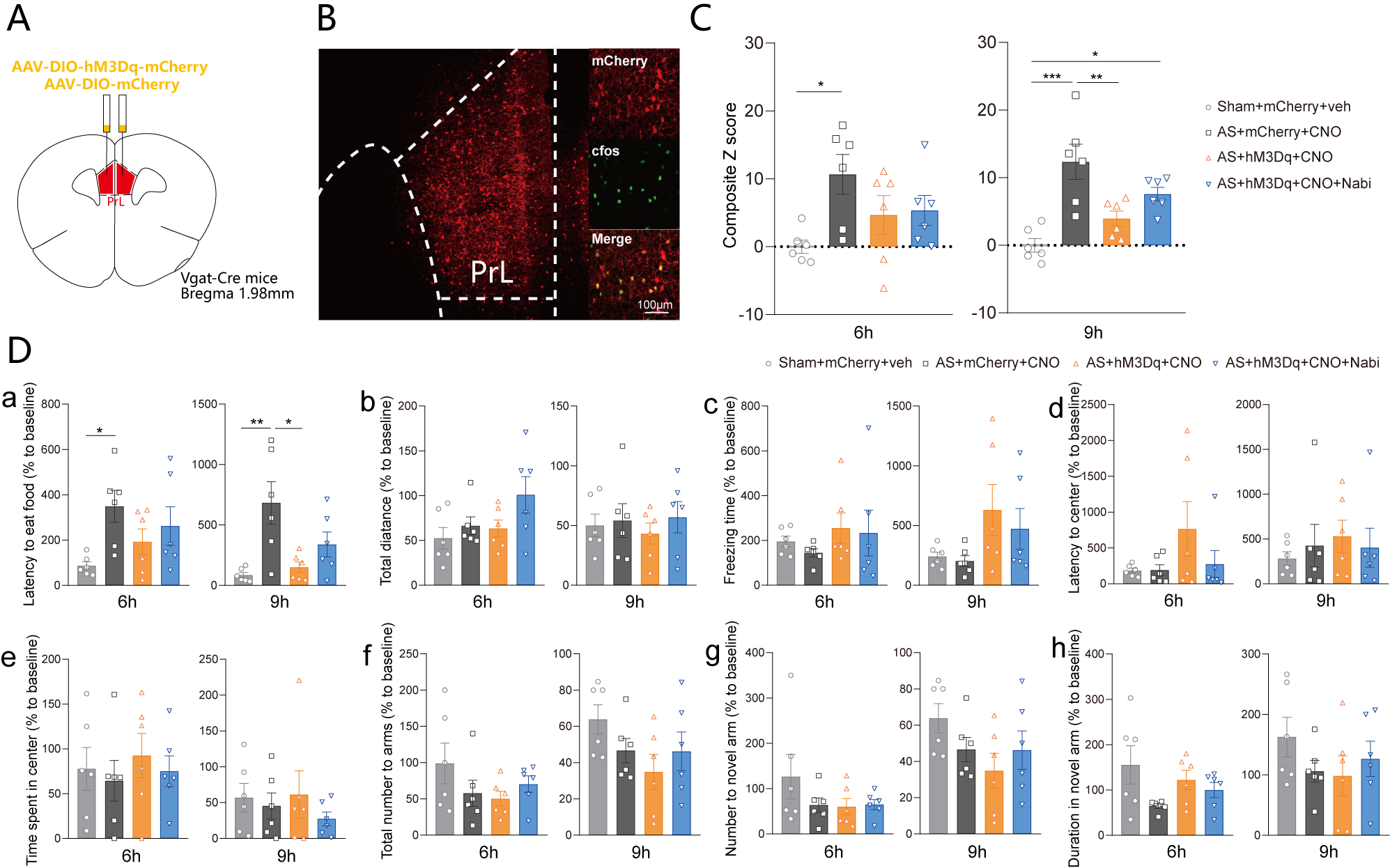

Supplement: Supplementary Figure 2 — PrL GABAergic neurons modulated POD without responses to cannabinoid. (A) Scheme of viral injection. (B) The injection site (left) and activation efficiency (right). (C) The composite Z score at 6 h (left) and 9 h (right) after AS. (D) The latency to eat food in buried food test (a); the total distance (b), the freezing time (c), the latency to center (d), time spent in center (e) in open field; the total number to arm (f), number to novel arm (g), duration in the novel arm (h) in Y maze at 6 h (left) and 9 h (right) after AS. Data are shown as mean ± SEM. N = 6. *P < 0.05, **P < 0.01, ***P < 0.001. ISO, isoflurane; AS, anesthesia & surgery; Nabi, nabilone; CNO, clozapine N-oxide; mCherry, AAV–DIO-mCherry; hM3Dq, AAV-DIO-hM3Dq-mCherry; PrL, prelimbic cortex. [file Image_2.TIF]

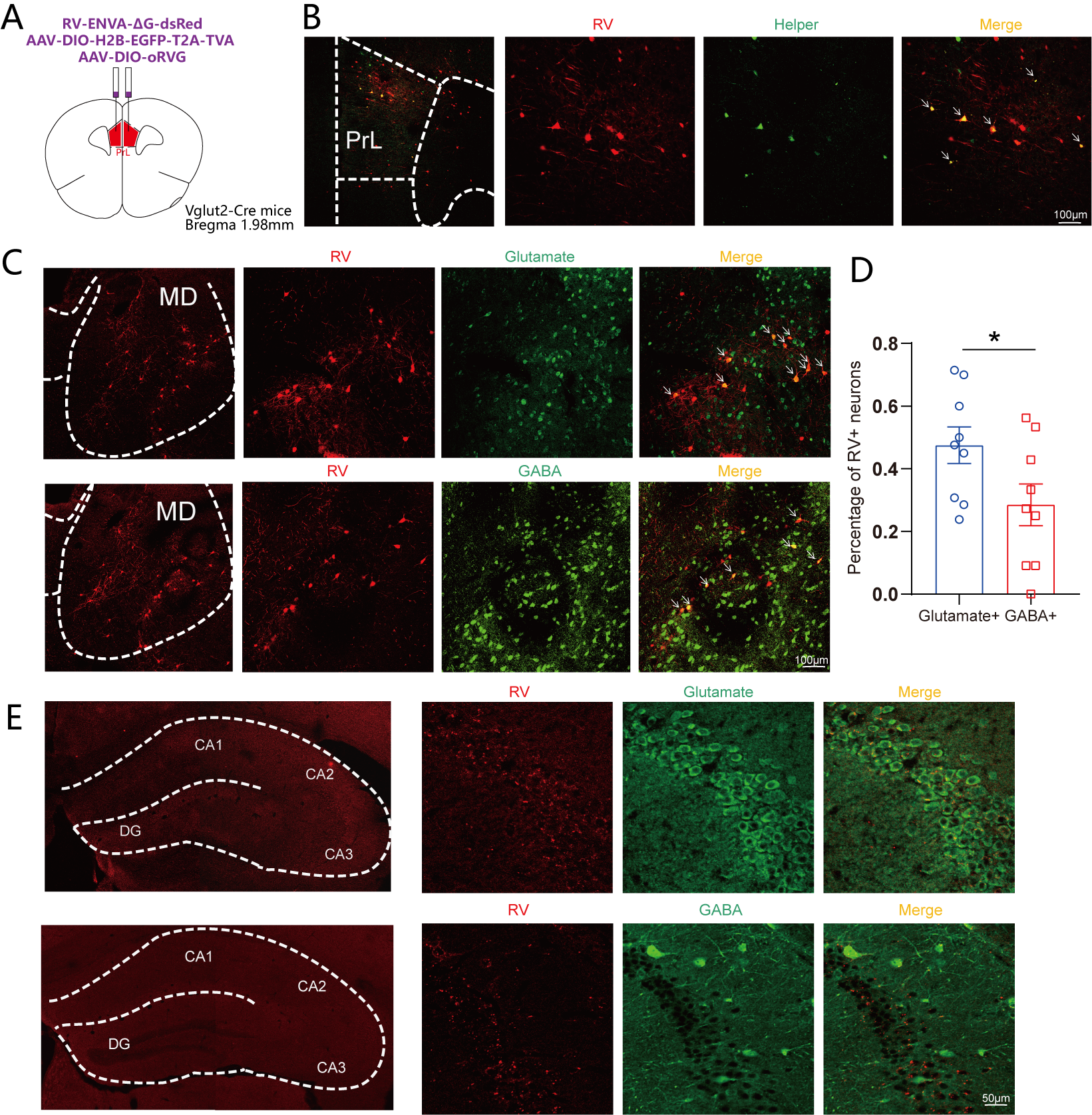

Supplement: Supplementary Figure 3 — The RV retrograde labeled neurons in the MD and HIP from the PrL glutamatergic neurons. (A) Scheme of injection of helper and RV viruses into the PrL of Vglut2-Cre mice. (B) The expression of the viruses in the PrL. (C) The co-localization of RV-labeled neurons and glutamatergic and GABAergic neurons in the MD. Arrowheads indicate the co-labeled cells. (D) The percentage of glutamatergic (blue bar) and GABAergic neurons (red bar) in the RV-labeled neurons. (E) The co-localization of RV-labeled neurons and glutamatergic and GABAergic neurons in the HIP. Data are shown as mean ± SEM. N ≥ 3. *P < 0.05, ****P < 0.0001. RV, RV-ENVA-ΔG-dsRed; Helper, AAV-DIO-H2B-EGFP-T2A-TVA & AAV-DIO-oRVG; PrL, prelimbic cortex; MD, mediodorsal thalamus. [file Image_3.TIF]

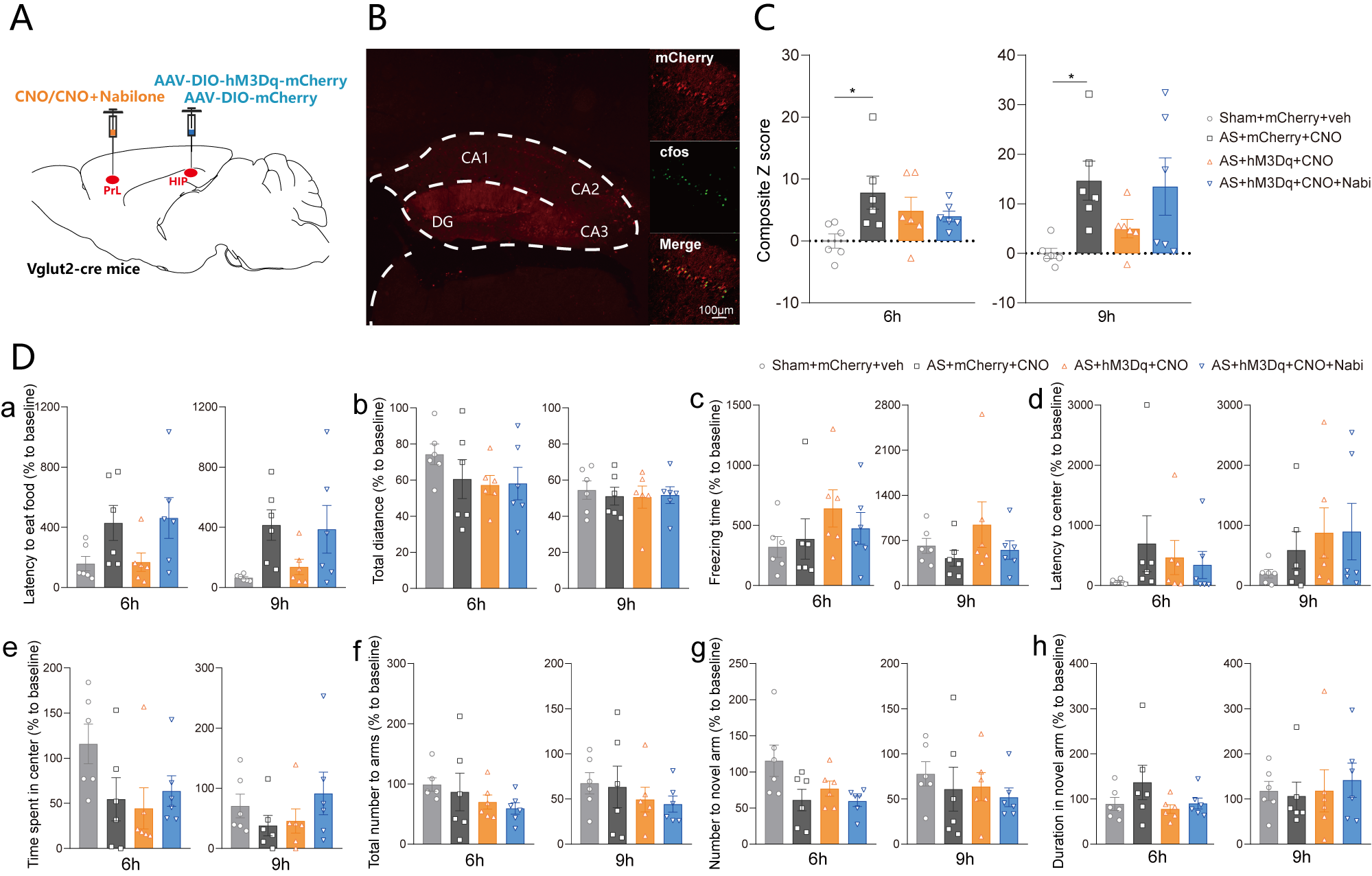

Supplement: Supplementary Figure 4 — Activation of HIPglu - PrL circuit did not affect POD. (A) Scheme of viral injection and drug application. (B) The injection site (left) and activation efficiency (right). (C) The composite Z score at 6 h (left) and 9 h (right) after AS. (D) The latency to eat food in buried food test (a); the total distance (b), the freezing time (c), the latency to center (d), time spent in center (e) in open field; the total number to arm (f), number to novel arm (g), duration in the novel arm (h) in Y maze at 6 h (left) and 9 h (right) after AS. Data are shown as mean ± SEM. N = 6. *P < 0.05. AS, anesthesia & surgery; Nabi, nabilone; CNO, clozapine N-oxide; mCherry, AAV-DIO-mCherry; hM3Dq, AAV-DIO-hM3Dq-mCherry; PrL, prelimbic cortex; HIP, hippocampus. [file Image_4.TIF]
